# Health-Promoting Properties of *Lacticaseibacillus paracasei:* A Focus on Kefir Isolates and Exopolysaccharide-Producing Strains

**DOI:** 10.3390/foods10102239

**Published:** 2021-09-22

**Authors:** Ana Agustina Bengoa, Carolina Dardis, Graciela L. Garrote, Analía G. Abraham

**Affiliations:** 1 Centro de Investigación y Desarrollo en Criotecnología de Alimentos (CIDCA, UNLP-CIC-CONICET), La Plata 1900, Argentina; bengoaagustina@gmail.com (A.A.B.); carolinadardis@gmail.com (C.D.); ggarrote@biol.unlp.edu.ar (G.L.G.); 2 Área Bioquímica y Control de Alimentos, Facultad de Ciencias Exactas (UNLP), La Plata 1900, Argentina

**Keywords:** *Lacticaseibacillus paracasei*, kefir, bioactive compounds, exopolysaccharide

## Abstract

Among artisanal fermented beverages, kefir (fermented milk drink) and water kefir (fermented nondairy beverage) are of special interest because their grains can be considered natural reservoirs of safe and potentially probiotic strains. In the last years, several reports on *Lacticaseibacillus paracasei* (formerly *Lactobacillus paracasei*) isolated from both artisanal fermented beverages were published focusing on their health-promoting properties. Although this is not the predominant species in kefir or water kefir, it may contribute to the health benefits associated to the consumption of the fermented beverage. Since the classification of *L. paracasei* has been a difficult task, the selection of an adequate method for identification, which is essential to avoid mislabeling in products, publications, and some publicly available DNA sequences, is discussed in the present work. The last findings in health promoting properties of *L. paracasei* and the bioactive compounds are described and compared to strains isolated from kefir, providing a special focus on exopolysaccharides as effector molecules. The knowledge of the state of the art of *Lacticaseibacillus paracasei* from kefir and water kefir can help to understand the contribution of these microorganisms to the health benefits of artisanal beverages as well as to discover new probiotic strains for applications in food industry.

## 1. Introduction

Fermented foods have played an important role in the human diet since the development of civilization and remain an integral part of local culture and traditions in many countries. A positive relationship between the consumption of fermented dairy products, health status and the intestinal microbiota has been reported. Fermented foods can contribute to health benefits by providing the consumer with beneficial microorganisms and nutritional benefits associated to changes performed in the food matrix during fermentation [1]. Some artisanal fermented foods contain several taxa and even different strains, with the population’s dynamic during processing being remarkably complex [2].

Among artisanal fermented beverages, kefir (fermented milk drink) and water kefir also known as “aquakefir” or “sugary kefir” (fermented non-dairy beverage) are of special interest because of their natural and artisanal production, which is compatible with sustainable technology. They have a common characteristic, the starter used for their production are kefir grains or water kefir grains that contain a complex microbiota immobilized in a polymeric matrix [3,4].

Due to their complex microbiota, kefir grains or water kefir grains can be considered as natural reservoirs of safe and potentially probiotic strains. Studies on kefir grain microbiota revealed that *Lactobacillus kefiranofaciens*, *Lentilactobacillus kefiri* and *Lentilactobacillus parakefiri* are the most representative species; however, also other species are described, including *Lacticaseibacillus* *paracasei*, *Lactobacillus acidophilus*, *Lactobacillus delbrueckii* subsp. *bulgaricus*, *Lactiplantibacillus* *plantarum* and *Lactococcus lactis*. More than 23 species of yeast have been identified as part of the kefir grain microbiota. These include *Saccharomyces cerevisiae*, *S. unisporus*, *Candida kefyr* and *Kluyveromyces marxianus* [5,6].

Water kefir grains have been less studied than those isolated from kefir, although they are also an important reservoir of highly competitive microorganisms. Some of the key microorganisms of water kefir fermentation are *Lentilactobacillus hilgardii*, *Liquorilactobacillus nagelii*, *L. paracasei*, *Bifidobacterium aquikefiri*, *S cerevisiae* and *Dekkera bruxellensis* [7].

Kefir and sugary kefir are described to exert beneficial effects on consumers’ health. The most relevant scientific findings on health benefits associated with kefir consumption were summarized by several authors [3,4,8,9]. The health promoting properties of artisanal fermented beverages could be attributed to the diversity of microorganisms that they contain [10].

*Lacticaseibacillus paracasei* (formerly *Lactobacillus paracasei*) is one of the species isolated from both artisanal fermented beverage’s starters. Although it is not the predominant species in kefir or water kefir, it may contribute to their health-promoting properties. It belongs to the genus *Lacticaseibacillus* (formerly *Lactobacillus casei* group (LCG)), which is composed of the closely related species *L. casei*, *L. paracasei* and *L. rhamnosus*, among others and contains several strains with a long history of apparently safe use in food and agricultural applications that have been studied for their health-promoting properties [11,12,13].

The present review will discuss some issues relating to the probiotic properties of *L. paracasei* and their effector molecules, focused on the role of exopolysaccharides (EPS). A special emphasis on strains isolated from artisanal fermented beverages will be performed highlighting the relevance of these products as sources for new probiotic strains for applications in food industry. Along the review, the taxonomic and genetic aspects of *L. paracasei* strains will be considered as well as the role of effector molecules associated with health-promoting properties.

## 2. *Lacticaseibacillus paracasei*: A Tour through the Evolution of Taxonomical Classification

Due to the broad interest in *L. casei* group members for use in the food and pharmaceutical industries as probiotics, their genotypic and phenotypic properties have been extensively studied, and their classification and taxonomy have been discussed [14]. Unequivocally identification of *L. casei* group members has been a matter of extensive discussion since the initial description of the species by Collin et al. in 1989 [15]. Taking into consideration the heterogeneity in DNA–DNA hybridization assays and biochemical tests between different *L. casei* strains, the authors reclassified the group proposing a new species named *L. paracasei* with two subspecies *L. paracasei* subsp. *paracasei* and subsp. *tolerans*.

Since then, *L. paracasei* taxonomy has been convoluted together from the use of conventional phenotypic methods to the advance of molecular genotyping for determining evolution and phylogeny [14,16]. However, the consideration of *L. paracasei* as a novel species was first questioned on account of misunderstandings and discrepancies regarding the identification of the type strain [17,18,19,20]. Some authors suggested the rejection of the name *L. paracasei* and proposed that the strain designated as type strain *L. casei* ssp. casei ATCC 393 should, in fact, be reclassified as *L. zeae* and that *L. paracasei* strains should be reclassified under *L. casei*, with *L. casei* ATCC 334 as the neotype strain for this species [20].

However, these suggestions were not accepted by Judicial Commission of the International Committee on Systematics of Bacteria (2008) indicating that strain ATCC 393 was the type strain for *L casei*, rejecting the name *L. zeae* because it contravenes Rules 51b (1) and (2) of the International Code of Nomenclature of Bacteria and classifying ATCC 334 as a member of a different taxon named *L. paracasei* [21].

The advance in pan and core genome methods for determining evolutionary relationships provided new tools for taxonomy. An in-depth comparative genomic analysis of the *L. casei* group was performed by Wuyst et al. (2017) who proposed a classification of this group in three clades based on (a) differences in whole-genome Guanine and Cytosine (GC) content, (b) a core genome phylogenetic tree constructed on the alignment of 776 single-copy marker genes and (c) pairwise genome distances [14].

One clade represents the species of *L. rhamnosus*, including the type strain *L. rhamnosus* DSM 20021. The second clade contains *L. casei* and *L. paracasei* isolates and includes the *L. paracasei* type strain ATCC 334, while the third clade consist of *L. casei* and *L. zeae* isolates. However, since *L. zeae* isolates were reclassified as *L. casei*, this clade actually represents *L. casei* strains. Based on these results, most of the strains designated as either *L. casei* or *L. paracasei* subsp. *paracasei* in the literature are, in fact, in the same clade corresponding to the species named *L. paracasei*.

Salvetti et al. (2018) evaluated the relatedness of 269 species belonging primarily to the families Lactobacillaceae and Leuconostocaceae through the analysis of ribosomal proteins and housekeeping genes and the assessment of the average amino acid identity (AAI) and the percentage of conserved proteins (POCP) as the basis for reclassification [22].

**Figure 1:**
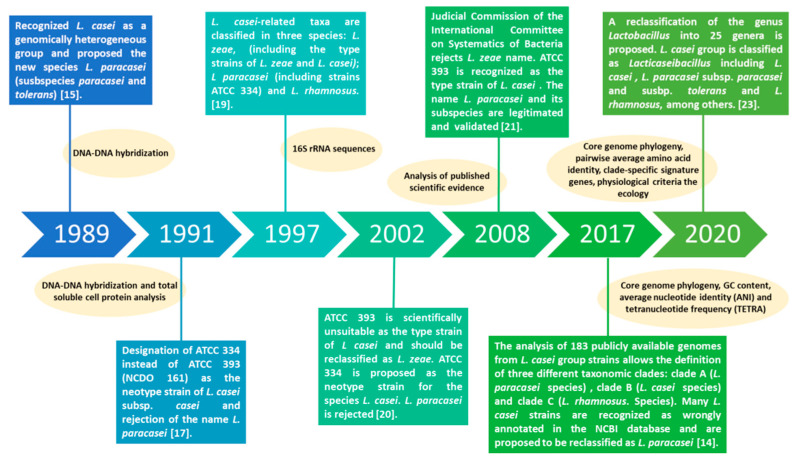
Timeline of the main events on evolution of classification and taxonomy of *Lacticaseibacillus* along with the methodology applied in each report. ATCC: American Type Culture Collection; NCDO. National Collection of Food Bacteria.

By using a polyphasic approach (core genome phylogeny, (conserved) pairwise average amino acid identity, clade-specific signature genes, physiological criteria and the ecology of the organisms), Zheng et al. (2020) recently recommended the reclassification of the genus *Lactobacillus* into 25 genera that reflects the phylogenetic position of the microorganisms but also considers the ecological and metabolic properties. This new classification proposed the genus *Lacticaseibacillus*, which includes the species *Lacticaseibacillus paracasei* to name the formerly called *Lactobacillus paracasei* [23]. Figure 1 summarizes the evolution of classification and taxonomy of *L. paracasei* over the years with a non-exhaustive list of reports published.

## 3. Identification and Classification of *Lacticaseibacillus paracasei*

Since the three species that belongs to the formerly called *L. casei* group are closely genotypically related, the selection of an appropriate methodology for their unequivocally classification is necessary [11].

The selection of an adequate method for identification is essential to avoid mislabeling in products, publications and some publicly available DNA sequences [11,24,25]. Heterogeneities found in the 16S rRNA genes of several strains of *L. casei*/*paracasei* was one of the difficulties encountered in obtaining clear-cut taxa within the *L. casei*/*paracasei* complex [26]. Furthermore, a response to niche adaptation led to loss of redundant genes followed by the acquisition of genes by horizontal gene transfer, which results in heterogeneity depending on the environment [24,27]. In fact, studies on *L. paracasei* strains isolated from cheese demonstrated that heterogeneities are also found even between strains isolated from the same niche [28].

Thus, several methods to unequivocally classify *L. paracasei* were proposed and were reviewed in detail [11,25]. Among them, phenotypic methods, such as a traditional fermentation test accompanied by a PCR (polymerase chain reaction) DNA-sequencing method for the analysis of 16S ribosomal RNA (16S rRNA) genes were some of the first methods proposed for classification of the *L. casei* group [19,29]. Iacumin et al. (2015) proposed the use of species-specific PCR and high-resolution melting (HRM) analysis. They applied these methods to evaluate 201 strains from international culture collections belonging to the *L. casei* group and found that 46 strains were different from their previous classification [30]. Huang and Lee (2011) proposed used *dnaK* sequencing to complement 16S rRNA for the classification of the *L. casei* group [31]. Multiplex PCR assay based on *mutL* was used to effectively distinguish *L. casei*, *L. paracasei* and *L. rhamnosus* [32].

Wuyst et al. (2017) performed a comparative analysis of 183 public available *L. casei* group genome assemblies and also found some inconsistencies in the taxonomy and reclassified all of the genomes accordingly [14]. Based on their results, the authors suggested the use of whole-genome ANI compared to *L. casei* group type strains to differentiate between the species *L. casei* and *L. paracasei* [14]. They also suggested that the use of the heme-dependent catalase or the superoxide dismutase gene as marker genes could be used for correct differentiation of these species if sequencing of the 16S rRNA gene leads to the identification of a member of the species *L. casei*/*L. paracasei*.

Huang and Huang (2018) successfully differentiated *L. paracasei* strains even into subspecies by using level matrix-assisted laser desorption/ionization time-of-flight mass spectrometry (MALDITOF MS) and an analytical in-house database (IHDB) accompanied with bioinformatics tools [33]. They used housekeeping gene sequencing and species-specific PCR to validate the MALDI-TOF MS platform. Housekeeping genes, such as the phenylalanyl t-RNA synthase alpha subunit (*pheS*) and RNA polymerase alpha subunit (*rpoA*), were also used to distinguish closely related species [34] as well as multilocus sequence analysis (MLSA) based on concatenated sequences of three housekeeping genes (*dnaK*, *pheS* and *yycH*) [35].

Silveraju et al. (2020) evaluated the *pheS* genes from a total of 266 type strains of lactobacilli for which genomes are publicly available. The extracted *pheS* sequences were compiled in a database (*pheS*-DB) to allow a comprehensive and confident characterization of the community diversity and structure of lactobacilli at the species level [36]. According to Huang et al. (2018) combining species- and subspecies-specific identification methods, housekeeping gene sequences and MALDI-TOF MS spectral pattern analysis resulted as quick and accurate methods for identifying strains in the *L. casei* group at the species and subspecies levels [25]. However, it should be taken into consideration that tests based on phenotypic markers should be performed under strictly controlled growth conditions.

Methods used for identification of *L. paracasei* strains isolated from traditional kefir or water kefir also varied. Most of them used 16S rRNA gene sequencing alone [37,38,39] or 16S rRNA gene sequencing accompanied by other methods, such as the use of species-specific primers [40,41] or ribosomal DNA restriction analysis (ARDRA), Repetitive Element Palindromic PCR (REP-PCR) fingerprint and Random Amplified Polymorphic DNA (RAPD) [42]. MALDI-TOF (Matrix-assisted laser desorption ionization time-of-flight) mass spectrometry was also used for the identification of strains isolated from kefir [43] as well as a polyphasic approach combining phenotypic and genotypic methods based on (GTG)5-R fingerprint and sequence analysis of the housekeeping gene encoding the α-subunit of bacterial phenylalanyl-tRNA synthase (*pheS*) [44]. Recently, Kim et al. (2020) performed a comparative genomic analysis of the pan genome and core genome of *L. casei* group species to designed species-specific primers for a differential identification of *L. paracasei* with real time PCR [45].

The diversity of methods developed for the identification of *L. paracasei* indicates that its differentiation from the closely related species is a difficult task and that a combined approach should be performed for an unequivocally classification. However, the advance in genome analysis contributes to the better understanding of *L. paracasei* phylogenetic relationship that would improve the selection of identification methods.

## 4. *Lacticaseibacillus paracasei* Health-Promoting Properties and the Effector Molecules Associated

The mechanisms related to the health effects displayed by probiotics (live microorganisms that, when administered in adequate amounts, confer a health benefit on the host [46]) are of interest among researchers. Probiotics can affect human/animal health in various ways, such as inhibiting intestinal pathogenic microorganisms, modulating the immune response, reducing serum cholesterol concentration, or exerting antioxidant activity, among others. Such effects may be due to the presence of the microorganism itself or to metabolites that it produces and that in certain cases releases into the environment (bacteriocins, exopolysaccharides and organic acids, for example).

Many are the benefits reported in the literature for *L. paracasei* strains [11,47]. These include antimicrobial and antibiofilm activity; immune system stimulation; anti-inflammatory, antioxidant, anti-obesity and anti-proliferative/proapoptotic, lipid metabolism improving, hypocholesterolemic and stress modulator effects; and the enhancement of intestinal bacterial microbiota; among others. Health promoting properties can be ascribed to the consumption of the viable microorganisms that generate molecules in situ that also contribute to well-being, as well as the metabolites produced during fermentation are associated with the changes made in the matrix of the foods that contain them.

Given that most effects of probiotics are strain-specific [48], the intrinsic high heterogeneity existing among *L. paracasei* strains makes this species an optimal source for the selection of novel candidate probiotic strains possessing unique technological and health-promoting traits [49]. Table 1 lists the most recent reports on health functional properties attributed to different strains isolated from different sources with a special focus on kefir and other traditional fermented products indicating if the study was performed with whole product, microorganisms or cell free supernatant.

Among *L. paracasei* that have been studied for their beneficial effects on consumer health, there are several isolates from kefir of Argentina, Brazil, Greece, Iran, Russia and Tibetan grains and water kefir from Mexico and Belgium. Most of the strains were obtained from kefir and only a few from water kefir. Protection against pathogens, immunomodulation and anti-inflammatory, prebiotic, antioxidant and antiproliferative activity are the beneficial properties that have been attributed to these microorganisms.

What is the mechanism by which *L. paracasei* confers health benefits on the host? What are the molecules involved in such effects? Although in recent years much effort has been made to study this, the answer is not conclusive, and there remains much to be elucidated. In some studies, a biological effect mediated by the viable microorganism was evidenced (indicated in green in Table 1), while, in other cases, the inanimate microorganisms and/or their components, known as postbiotics [72,73] as well as their metabolites, were responsible for the beneficial effect on health (indicated in yellow in Table 1). On certain occasions, an effect due to the whole fermented product (marked in light blue in Table 1) was described, and, in several reports, a beneficial effect of the EPS was demonstrated (marked in pink in Table 1).

As mentioned above, the probiotic effect of *L. paracasei* strains isolated from kefir could be associated with the presence of the microorganism. For example, viable whole cells of *L. paracasei* CIDCA 8339, CIDCA 83123 and CIDCA 83124 isolated from Argentinean kefir grains have the capacity to adhere to epithelial intestinal cells and protect against *Salmonella* invasion as well as immunomodulate and protect the gastric epithelium [64,66,74]. In the same direction, Karaffová et al. (2021) described that the strain I2 isolated from Tibetan kefir grains increased the proportion of all T cells (CD3^+^), CD4^+^ lymphocytes and the ratio of CD4^+^:CD8^+^ cells in vivo and increase the gene expression for mucins at the local intestinal level [43].

Otherwise, the probiotic effect of *L. paracasei* strains isolated from kefir could be associated with an effector molecule. The evidence provided in recent years has led to the evaluation of lactic acid as a health-promoting agent in fermented foods [75]. This acid, as the main product of the metabolism of lactic acid bacteria, influences its probiotic action, playing an important role both in the control of pathogens and in the immunomodulatory activity. It has been demonstrated that lactate can regulate critical functions of macrophages and dendritic cells and modulate the inflammatory activation of epithelial cells as well.

In addition to the organic acids consumed with the fermented foods, the acids produced by lactobacilli in situ can contribute to the probiotic action. It was described that the *L. paracasei* strains CIDCA 8339, 83123 and 83124 and isolated from kefir can adhere to Caco-2 cells and mucin after passage through simulated gastrointestinal tract [74] giving to the lactobacilli the possibility to produce lactate in the gut epithelium microenvironment. Intraluminal levels of lactate derived from fermentative metabolism of lactobacilli were shown to modulate the inflammatory response in the intestinal mucosa.

Furthermore, lactate can be used by the gut microbiota to produce acetate, propionate and butyrate; short chain fatty acids (SCFA) highly associated to gut health that have been proven to down regulate the pro-inflammatory responses in intestinal epithelial and myeloid cells [76,77]. Among the molecular mechanisms responsible for these functions, histone deacetylase dependent-modulation of gene expression and signaling through G-protein-coupled receptors have been described [77]. In addition, Bengoa et al. (2020) demonstrated that the in vivo gastroprotective effect exerted by *L. paracasei* CIDCA 8339 involves not only the direct interaction of the microorganism with the gastric mucosa but also the in situ production of other metabolites that could modulate the inflammatory response [66].

Regarding other effector molecules described for kefir isolated *L. paracasei* strains, exopolysaccharides, bacteriocins and other metabolites can be mentioned. Miao et al., 2014 reported a bacteriocin F1 produced by *L. paracasei* FX6 strain with a wide antimicrobial spectrum [38], and Leite et al. (2015) detected a bacteriocin-like inhibitory substance produced by *L. paracasei* MRS59 strain from Brazilian kefir grains [42].

Recently, Duan et al. (2020) found an antimicrobial substance from *L. paracasei* FX6 that was able to interfere with important cellular functions through affecting the synthesis of protein and binding to genomic DNA [69]. Even more, Ghane et al. (2020) showed that neutralized cell-free supernatant of *L. paracasei* LAB2 and LAB4 strains inhibited the growth and the biofilm formation by uropathogenic *E. coli* [37].

On the other hand, a relevant antimicrobial, antifungal and antioxidant capacity of cell-free supernatant of *L. paracasei* CT12, an isolate from Mexican water kefir grains, was described by Romero-Luna et al. (2020) [39]. Additionally, a significant reduction of cancer-cell proliferation in vitro was evidenced with the cell-free supernatant of *L. paracasei* SP5, isolated from Russian kefir grains [40] and *L. paracasei* AGR4 isolated from Greece kefir grain [41]. However, the nature of the compounds responsible for these effects has not been yet determined.

## 5. Exopolysaccharides as Effector Molecules Associated to *Lacticaseibacillus paracasei* Health Benefits

As mentioned above, one of the effectors molecules ascribed to the health-promoting properties of *L. paracasei* are extracellular polysaccharides, which can be secreted to the environment in the form of slime EPS or can be attached to the cell surface in the form of capsule (CPS) [78]. According to their structure, EPS are classified into two groups: homopolysaccharides (HoPS) and heteropolysaccharides (HePS) [79]. The first ones are composed of a single type of monosaccharide either glucose (α-glucans and β-glucans) or fructose (β-fructans).

Meanwhile, heteropolysaccharides, which constitute the majority of EPS produced by lactic acid bacteria (LAB), are composed of a backbone of repeating subunits branched or unbranched that consist of three to eight monosaccharides, commonly rhamnose, fructose, galactose or glucose. In some cases, HePS could contain some modifications, such as acetylations, pyruvylations and phosphorylations [80,81,82]. There are substantial differences in the number of enzymes and steps implied in the synthesis and export out of the cell between HoPS and HePS.

HoPS are synthetized completely outside the cell by the activity of an extracellular glycosyltransferase that uses sucrose as a substrate. Instead, HePS biosynthesis is a much more complex process. First, the cell must internalize monosaccharides and disaccharides from the growth media through the PEP-PTS system, although this can also occur by active transport. Once inside the cell, the sugar can be used in catabolic pathways (as sugar-6-phosphate) or for polysaccharide synthesis (as sugar-1-phosphate). Before polymerization begins, the sugar is activated by the addition of uridine diphosphate (e.g., UDP-glucose and UDP-galactose) or deoxithymidine diphosphate (e.g., dTDP-rhamnose), thus, forming sugar nucleotides.

Then, a priming glycosyltransferase (EpsE) binds the first sugar to a lipid carrier, followed by the action of other glycosyltransferases that continue with the saccharide unit formation. Finally, the undecaprenol-lipid bound with the repeated unit is translocated through the membrane by a flippase, the repeated units are joined by a polymerase, and other enzymes control the chain length and remove the lipid carrier [83].

Genes involved in EPS synthesis in different LAB strains were usually designated by alphabetical order of occurrence in a given locus [83]. In this way, genes codifying for different kind of enzymes were named equally leading to confusion in understanding the genes involved in EPS synthesis of different strains. Zeidan et al. (2017) described a general cluster structure for HePS synthesis in LAB, which was organized with five conserved genes at the 5′ end (*epsABCDE*) followed with a variable region that contains the flippase (*wyx*), polymerase (*wzy*) and glycosyltranfesases [83].

EPS clusters are conformed by four groups of genes that codify the proteins involved in assembly, modulation, glycosyltransferases and NDP-sugar biosynthesis. The enzymes involved in sugar nucleotides formation and monosaccharide decoration are not exclusively of polysaccharide synthesis pathway and can be codified within EPS cluster or not [83,84].

Smokvina et al. (2013) analyzed the pangenome, core genome and variable genome from *L. paracasei* by sequencing 34 *L. paracasei* strains from different niches and using complete genome sequences of three reference strains [27]. From this study, they found four different EPS clusters that were either chromosomally or plasmid encoded. EPS-1 or EPS-2 clusters were not detected simultaneously in the *L. paracasei* genomes studied. The third EPS cluster (EPS-3) was identified in most of the studied strains (29), and EPS-4 was detected in only one strain.

The presence of EPS-1 and EPS-3 suggests that rhamnose is an important constituent of EPS in *L. paracasei* strains since genes that encode rhamnosyltransferases and enzymes that catalyze the conversion of D-glucose-1-phosphate into dTDP-L-rhamnose are codified in those clusters [27]. Within the genome of *L. paracasei* DG, two EPS clusters were identified. Only one of them, EPS-a, was conserved in other five *L. paracasei* strains and *L. casei* Shirota. EPS-b, contained about 14 EPS-related genes, of which, 7 were only present in the DG strain and codified for putative glycosiltransferases, a flippase and a phosphotransferase [49].

Two EPS clusters were codified in the chromosome of *L. paracasei* IJH-SONE68 [61], while the LC2W strain presented only one EPS cluster with a similar structure to the *cps* cluster described in *L. casei* Shirota [85,86]. Although the EPS clusters of the mentioned *L. paracasei* strains presented certain differences in their genetic organization, most of them presented genes that encode enzymes that catalyze the conversion of D-glucose-1-phosphate into dTDP-L-rhamnose suggesting that rhamnose is an important constituent of EPS in these strains.

Regarding the *L. paracasei* strains isolated from kefir there is no information about the number of EPS clusters or the organization of the genes involved in EPS production. Up to the present, the complete genomic sequence of only one *L. paracasei* strain isolated from Tibetan kefir (submitted by Nanjing Agricultural University, Nanjing, Jiansu, China) named ZY-1 was available in the NCBI (National Center for Biotechnology Information) database and appears to be compound by the chromosome and four plasmids (pLPZ1-4).

Although the physiological role of EPS in the bacteria is not completely understood, it is thought to play an important role in the protection of microbial cells against harsh factors and conditions, such as toxic metals, innate immune factors, phage attack, osmotic stress and desiccation [81,82]. Moreover, this EPS layer could provide additional protection against low pH, bile salt, gastric and pancreatic enzymes, thus, guaranteeing the survival of orally administrated probiotics through the gastrointestinal tract [81].

In this context, it was demonstrated that the removal of the slightly attached EPS through successive washes with PBS led to a reduction in the tolerance of *L. paracasei* CIDCA 8339, CIDCA 83123 and CIDCA 83124 isolated from kefir grains to gastrointestinal conditions, supporting the protective role of EPS against stress conditions [65]. Similarly, Stack et al. (2010) demonstrated that the beta-glucan-producing *L. paracasei* NFBC 338 isolated from the human gastrointestinal tract showed higher tolerance to both technological and gastrointestinal stresses than the original non beta-glucan-producing strain [87].

Moreover, this surface polymer is involved in aggregation, biofilm formation and interaction of the lactobacilli with intestinal epithelial cells [81,88]. EPS from LAB are surface molecules that directly interact with intestinal mucosa exerting strain-dependent negative or positive effects in adhesion [89]. The removal of the EPS layer from *L. paracasei* DG did not significantly affect its adhesion to Caco-2 cells [49].

Otherwise, *L. paracasei* CIDCA 8339, CIDCA 83123 and CIDCA 83124 increased their ability to adhere to mucin and intestinal epithelial cells in vitro after the passage through gastrointestinal conditions that could be ascribed to induction of EPS synthesis. Proteomic analysis suggested that gastrointestinal stress induced changes in the expression of enzymes involve in EPS production, which could be responsible for the differences observed in the adhesion properties of lactobacilli [74].

Similarly, when comparing the adhesion ability of EPS-producing *L. paracasei* subsp. *paracasei* BGS J2-8 isolated from natural dairy product with its non-EPS producing mutant to Caco-2 and HT-29 intestinal cells, it was observed that the absence of EPS led to a significant reduction in the adhesion to both cells lines [88]. Probiotics, which are usually administered orally, must survive the gastrointestinal conditions in order to reach and colonize the gut. In this context, the production of EPS could imply an important advantage for potentially probiotic strains to display their health effects in situ.

In addition to their exceptional physicochemical properties, EPS present in foods constitute relevant bioactive metabolites with underlying biological activities that significantly contribute to the healthy properties of the final product [90]. The biological activities evidenced with *L. paracasei* EPS included antioxidative, anti-bacterial, cholesterol-lowering, immunoregulatory, anti-tumor, anti-inflammatory and prebiotic properties (Table 1).

High levels of certain blood lipids represent a risk factor for the development of cardiovascular diseases, one of the major causes of deaths worldwide [82]. In this context, the use of probiotics or probiotics’ metabolites, such as EPS emerge as an alternative to reduce blood cholesterol levels through diet. Bhat et al. (2019) demonstrated the cholesterol-reducing potential of EPS synthetized by *L. paracasei* M7, a strain isolated from breast milk.

From the hypocholesterolemic effect, this EPS displayed antioxidant and hydroxyl radical scavenging activity [58]. In the same direction, the EPS synthetized by *L. paracasei* subsp. *paracasei* NTU 101, a strain isolated from human feces, showed potential antioxidant activity evidenced by its ability to reduce linoleic acid peroxidation, its reducing power and its chelant and free radical scavenging activity [59].

On the other hand, the induction of oxidative stress and the consequent endoplasmic reticulum stress mediated by *L. paracasei* subsp. *paracasei* M5L EPS is responsible for the apoptotic effect attributed to this biomolecule on human colorectal adenocarcinoma cell line HT-29 [60]. Considering this, EPS that induced ROS production in malignant cells could eject a beneficial effect in patients that suffer from cancer diseases as a complement to traditional therapy.

The ability of EPS from *L. paracasei* strains to prevent adhesion to intestinal cells [88] and to reduce biofilm formation of pathogenic bacteria [58] has also been reported in the literature contributing to the treatment and prevention of infectious diseases produced by biofilm-forming pathogens [91].

Moreover, some EPS have the ability to interact with the immune system. However, the broad structural diversity among EPS may influence their recognition by receptors of the immune system resulting in different effects. On the other hand, Liu et al. (2011) demonstrated that *L. paracasei* subsp. *paracasei* NTU 101 EPS stimulated the growth of murine macrophages Raw 264.7 and increased the levels of pro-inflammatory molecules, such as IL-1 β, TNF α, IL-6 and nitric oxide, which consequently led to an induction of phagocytic activity [59].

Similarly, the rhamnose-rich EPS synthetized by *L. paracasei* DG enhanced the expression of pro-inflammatory cytokines TNF α, IL-6 and the chemokines IL-8 and CCL20 in human monocytic cell line THP-1, ejecting an immunostimulatory effect [49]. On the contrary, Noda et al. (2018) demonstrated the in vitro the anti-inflammatory effect of *L. paracasei* IJH SONE 68 EPS (isolated from fig leaf) mediated by the inhibition hyaluronidase [61].

Furthermore, the authors studied the potential used of this EPS as anti-allergenic against contact dermatitis using a picryl-chloride-induced delayed-type (type IV) allergy mice model. An anti-allergic effect was evidenced through oral administration as well as topic administration of this EPS, being the last effect likely mediated by the inhibition of the hyaluronidase activity but the mechanism is not described [62].

The prebiotic effect of EPS from *L. paracasei* strain has also been evidenced. Prebiotics are defined as “substrates that are selectively utilized by host microorganisms conferring a health benefit” [92]. Due to their complex structure, EPS produced by LAB can resist gastric and intestinal degradation reaching the intestinal level were they act as a substrate for commensal bacteria, stimulating the development of beneficial microorganisms and the production of bioactive metabolites, such as lactate and SCFA, which are involved in different cell mechanisms that play a key role in host health [81,82].

Traditionally, the targets of prebiotic action were mainly *Lactobacillus* and *Bifidobacterium*, genera with recognized probiotic properties [93]. Sarikaya et al. (2015) demonstrated that *L. paracasei* subsp. *paracasei* LB-8 EPS has the ability to modulate the intestinal microbiota by promoting the growth of *Bifidobacterium breve* in levels comparable to that of the known prebiotic inulin [63]. However, currently, the concept of prebiotics has expanded to include the modulation of other genera, in particular, those involved in the production of SCFA, such as *Eubacterium*, *Propionibacterium*, *Roseburia* and *Akkermansia*, among others [92,93].

**Figure 2:**
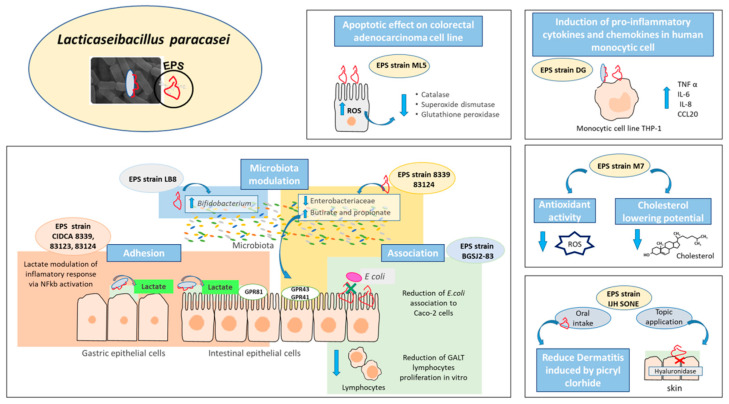
Beneficial effects and mechanisms ascribed to EPS isolated from *L. paracasei* strains. At the gastrointestinal level, EPS produced by the BGSJ2-83 strain reduced the association of *E. coli* to Caco-2 cells and reduced GALT lymphocyte proliferation in vitro. EPS from the LB8 strain modulated the microbiota composition due to a bifidogenic effect; meanwhile, EPS from CIDCA 8339 and CIDCA 83124 strains reduced Enterobacteriaceae. In addition, EPS isolated from CIDCA 8339 and 83124 produced a change in the concentration of short chain fatty acids by increasing the butyrate and propionate levels. EPS is involved in the bacteria association to epithelial cells where the in situ production of lactate modulated the inflammatory response via NFkb activation. Additionally, EPS from MLS strain exerted an apoptotic effect on the human colorectal adenocarcinoma cell line HT-29. The oral intake of EPS produced by IJH-SONE reduced dermatitis induced by picryl-clorhide, and the topical application of EPS reduced the inflammation in mice. EPS produced by the DG strain induced pro-inflammatory cytokines and chemokines in the human monocytic cell line THP-1. EPS isolated from M7 strain presented anti-inflammatory activity and reduced cholesterol levels. ROS: reactive oxygen species.

The prebiotic potential of EPS synthetized by *L. paracasei* CIDCA 8339 and CIDCA 83124 has been studied in vitro evidencing that both polymers are metabolized by infant fecal microbiota leading to changes in the fecal microbiota profile and enhancing the production of SCFA, such as propionic and butyric acids [67]. In this context, EPS produced by LAB constitute an interesting alternative to establish a widely diverse intestinal microbiota usually associated with a healthy state. Figure 2 summarizes the health-promoting effects ascribed to EPS produced by different *L. paracasei* strains.

As described above, many studies of EPS from *L. paracasei* strains are described in the literature. However, when it comes to strains isolated from kefir and water kefir, only a few reports have been published. These include the EPS-producing *L. paracasei* KL1-Liu strain isolated from Tibetan kefir that ejects a protective effect against *Salmonella pullorum* infection in chicks when administered in combination with *L. plantarum* Zhang-LL [68]. Nevertheless, it is not possible to attribute this health benefit exclusively to the strain or its EPS.

Although the EPS structure of this strain has been partly described [94], the EPS cluster genes organization has not been studied in detail yet. Similarly, *L. paracasei* CIDCA 8339, CIDCA 83120, CIDCA 83121, CIDCA 83123 and CIDCA 83124 (all EPS-producing strains isolated from kefir grains) significantly reduce the invasion of *Salmonella* to Caco-2/TC-7 intestinal epithelial cells. However, the role of EPS has not been elucidated.

Moreover, EPS from *L. paracasei* CIDCA 8339 and CIDCA 83124 have been characterized not only to define their biological activity (effect on the innate immune response and prebiotic effect) but also evidencing the role of the biopolymer in the protection of the probiotic against gastrointestinal conditions and its adhesion to intestinal cells. These properties are of great relevance for the maintenance of the viable microorganism close to the gastrointestinal epithelial cells to allow a deep cross talk between them producing in situ effectors molecules, such as lactate, bacteriocins and exopolysaccharides that may contribute to create a health-promoting microenvironment.

## 6. Conclusions

As was demonstrated by the literature reviewed, *Lacticaseibacillus paracasei* (formerly *Lactobacillus paracasei*) is one of the species isolated from artisanal fermented beverages, such as kefir (fermented milk drink) and water kefir (fermented non-dairy beverage), that is gaining attention to the scientific community as novel strains for application in the food industry. When a new isolate is obtained, the first challenge is the selection of an adequate method for identification to avoid mislabeling in products, scientific reports and the publication of DNA sequences. A special focus must be to unequivocally identify *L. paracasei* strains and differentiate from other species of the *Lacticaseibacillus* genus.

Recent advances in molecular data on the genes and their expression by using metagenomics, transcriptomics and metabolomics not only allow for a better understanding of the phylogeny of this group contributing to classification and identification but also the knowledge of the ability to synthesize molecules that could be involved in its probiotic function. The knowledge of the mechanisms involved in beneficial properties of *L. paracasei* strains from artisanal products, such as kefir or water kefir, would contribute to understand their role in the fermented beverage and to develop products with novel strains based on scientific evidence of their health-promoting effects.

As was described, biological effect may be due to the presence of either the viable bacteria non-viable bacteria/rest of cells (postbiotic), or metabolites released from *L. paracasei* into the environment. *L. paracasei* cell-free supernatants have immunomodulatory and gastroprotective properties ascribed to the presence of lactate, a common effector molecule of all fermented products.

In addition, the inhibitory properties against a range of pathogenic microorganisms could be exerted by the presence of lactate or owing to the production of bacteriocins by certain *L. paracasei* strains (strain-specific effectors). Health properties attributed to the presence of the viable microorganism could be associated to the molecules expressed on its surface. However, little information has been reported about the effector molecules associated with beneficial effects and/or the mechanisms of action.

Considering the revised reports, extracellular polysaccharides appear to be relevant effector surface molecules in *L. paracasei*. Although the study of EPS production genes and the biological activity from many *L. paracasei* strains is reported in the literature, it is noteworthy that little is known regarding the chemical characterization of these macromolecules as well as the relationship between their structure and technological and health functionality.

In light of the foregoing, it can be concluded that kefir grains and water kefir grains are significant sources for the isolation of new, safe *L. paracasei* strains. In particular, EPS-producing strains are very promising probiotics due to the double-benefit attributed to these polymers, both in helping the probiotic bacteria to colonize the gut and in acting as an effector molecule.

The present work emphasizes the relevance of isolating and investigating new EPS-producing *L. paracasei* strains from kefir and water kefir for their application in the development of new functional products, highlighting this as an emerging research field to explore in the future.

## Figures and Tables

**Table 1:** Health functional properties attributed to *Lacticaseibacillus paracasei* strains isolated from different sources.

*L. paracasei* Strain	Origin	Health Functional Properties	Biological Activity *	Reference
Lpc-37^®^	DuPont de Nemours, Inc. trademark	Stress, mood and well-being modulation	Reduction of perceived stress and improvement of biomarkers related to stress in a clinical trial.	[13]
GKS6	Healthy infant feces	Antioxidant activity	Delay in the aging process in mice by enhancement of antioxidants activity, resulting in lower oxidative damage.	[50]
KBL 382	Korean healthy feces	Anti-inflammatory activity—Microbiota modulation	Reduction of INF-γ, IL-4, IL-6, TNF and IL-17A levels and increase of anti-inflammatory cytokine IL-10 and CD4^+^CD25^+^Foxp3^+^ T regulatory cells in mesenteric lymph nodes levels. Improvement of cell tight junction and mucus thickness. Increase in bacterial diversity of fecal microbiota.	[51]
		Ameliorates atopic dermatitis—Immunomodulatory activity—Microbiota modulation	Decrease in T helper cytokines and increase IL-10 and TGF-β production in skin tissue. Increase in the proportion of CD4^+^ CD25^+^ Foxp3^+^ T regulatory cells in mesenteric lymph nodes and changes in the composition of gut microbiota of oral treated mice.	[52]
28.4	Oral cavity of a caries-free individual	Immunomodulatory activity	Bacteria cells have antifungal activity against planktonic cells, biofilms and persisted cells of *Candida auris*.	[53]
			Postbiotic elements (free-cell supernatant) inhibit *C. auris* in vitro and protect *Galleria mellonella* infected with *C. auris* enhancing its immune status.	
L1	Sweet potato sour liquid	Microbiota modulation	Increase in the abundance of functions related to carbohydrate and protein metabolism and fatty acid biosynthesis in the intestinal microbiota. Improvement in the growth performance of chicken.	[54]
FZU103	Traditional Hongqu rice wine	Improvement of lipid metabolism	Regulation of lipid metabolic pathways of pathogen free mice feed with high fat diet (fatty acid degradation, fatty acid elongation, unsaturated fatty acids biosynthesis, glycerolipid, glycerophospholipid and arachidonic acid metabolism, primary bile acid biosynthesis and riboflavin metabolism). Regulation of the expression of hepatic genes involved in lipid metabolism and bile acid homeostasis and promotion of fecal excretion of bile acids.	[55]
B-14	Traditional fermented dairy product	Antiproliferative—Proapoptotic effects	Downregulation or upregulation of key genes in the cell proliferation, cell survival and intrinsic and extrinsic apoptosis pathways.	[56]
ZFM54	New-born infant’s feces	Protection against foodborne pathogens	In vitro inhibition of *Salmonella typhimurium*, *Micrococcus luteus* and *Listeria monocytogenes* by production of a pore forming bacteriocin ZFM54.	[57]
M7	Human breast milk	Hypocholesterolemic—Antioxidant activity—Protection against pathogens	Antibiofilm potential of EPS against several human pathogens.	[58]
NTU 101	Human feces	Immunomodulatory—Antioxidant activity	Induction of pro-inflammatory molecules (nitric oxide, IL-6, TNFα and IL-1β) and phagocytic activity in murine macrophages Raw 264.7. Antioxidant activity (scavenging of 1,1-Diphenyl-2-picrylhydrazyl radicals, inhibition of linoleic acid peroxidation, reducing power, chelating ability on ferrous ions).	[59]
M5L	Kumiss	Antiproliferative activity	Apoptotic effect on human colorectal adenocarcinoma cell line HT-29 mediated by induction of oxidative stress and endoplasmic reticulum stress.	[60]
DG	Commercial product	Immunomodulatory activity	Induction of pro-inflammatory cytokines TNF α, IL-6 and the chemokines IL-8 and CCL20 in human monocytic cell line THP-1.	[49]
IJH SONE	Fig leaf	Anti-inflammatory activity	Anti-inflammatory effect mediated by inhibition of hyaluronidase activity.	[61]
		Antiallergenic activity	Antiallergenic effect evidenced by oral and topic administration against contact dermatitis in mice.	[62]
LB-8	Feces	Modulation of intestinal microbiota	Bifidogenic effect in vitro.	[63]
CIDCA 8339, 83120, 83121, 83123, 83124	Kefir grains (Argentine)	Protection against pathogens	Adhesion to Caco-2 cells and prevention of *Salmonella* association/invasion in vitro depending on surface properties of the strain.	[64]
CIDCA83123, 83124, 8339	Kefir grains (Argentine)	Immunomodulatory activity	Modulation of the intestinal epithelial innate immune response by viable whole cell.	[65]
		Immunomodulatory activity	Fermented milk supernatants downregulate the induced innate immune response in intestinal and gastric cells, with lactate as the metabolite responsible of this effect.	[12,66]
CIDCA 83124, 8339	Kefir grains (Argentine)	Microbiota modulation and changes in SCFA profile	EPS 8339 and EPS 83124 modify the microbiota by reducing the enterobacteria and increasing the production of propionic and butyric acid.	[67]
CIDCA 8339	Kefir grains (Argentine)	Gastroprotection	Adhesion to AGS gastric cell line. The strain consumption shows a gastroprotective effect in an acute gastritis murine model.	[66]
Ž2	Kefir grains (Tibet)	Immunomodulatory activity	Increase in the proportion of all T cells (CD3^+^), CD4^+^ lymphocytes and the ratio of CD4^+^:CD8^+^ cells in vivo and increase in the gene expression for mucins (MUC-1 and MUC-2) and IgA at intestinal level.	[43]
KL1-Liu	Kefir grains (Tibet)	Protection against pathogens	Mixed probiotic of *L. paracasei* KL1-Liu (EPS producer) and *L. plantarum* Zhang-LL reduces the mortality of pullorosis in chicks.	[68]
MRS59	Kefir grains (Brazil)	Adhesion to intestinal epithelial cells	Adhesion to human Caco-2 epithelial cells, bacteriocin production.	[42]
		Antimicrobial-Antioxidant activity	Antagonistic activity against food pathogens by bacteriocin-like inhibitory substance and antioxidative activity of cell extract.	
FX6	Kefir grains (Tibet)	Antimicrobial activity	Bacteriostatic effect on *Pseudomonas putida* due to the increase of bacterial membrane permeability and ability of the antimicrobial substance to affect the synthesis of protein and bind to genomic DNA.	[69]
		Antimicrobial activity	Bacteriocin F1 with a wide antimicrobial spectrum.	[38]
		Antimicrobial activity	Antibacterial effect on *Serratia* in chicken breast during refrigerated storage.	[70]
LAB2, LAB4	Kefir grains (Iran)	Protection against pathogens	Neutralized cell-free supernatant inhibits the growth and the biofilm formation by uropathogenic *E. coli*.	[37]
SP5	Kefir grains (Russia)	Antiproliferative activity	Reduction of cancer cell proliferation in vitro in a time- and concentration-dependent manner.	[40]
SP5		Antioxidant activity	Fermentation metabolites produced by the breakdown of anthocyanins and other larger-in-size phenolic compounds present in chokeberry juice, leading to increased levels of total phenol content.	[71]
AGR4	Kefir grains (Greece)	Antiproliferative activity	Time- and dose-dependent antiproliferative activity of HT-29 cells and human melanoma cell line A375.	[41]
LMG R40086, LMG R39998, LMG R40122, LMG R40006	Water kefir grains(Belgium)	Adhesion to intestinal epithelial cells	Adhesion ability to Caco-2 cells.	[64]
CT12	Water kefir grains (Mexico)	Antimicrobial-Antioxidative activity	Antimicrobial, antifungal and antioxidant capacity of cell-free supernatant.	[39]

* Colors indicates that assays were performed with whole *L. paracasei* cell (green), cell-free supernatant or non-viable cells (yellow), whole fermented product (light blue) and EPS fraction (pink).

## Data Availability

Not applicable.
